# Modulation of a decision-making process by spatiotemporal spike patterns decoding: evidence from spike-train metrics analysis and spiking neural network modeling

**DOI:** 10.1186/1471-2202-14-S1-P10

**Published:** 2013-07-08

**Authors:** Laureline Logiaco, René Quilodran, Wulfram Gerstner, Emmanuel Procyk, Angelo Arleo

**Affiliations:** 1 CNRS - UPMC Univ P6, Laboratory of Neurobiology of Adaptive Processes, UMR 7102, Paris, 75005, France; 2 Oficina de Educación Médica, Facultad de Medicina Universidad de Valparaiso, Hontaneda 2653, Valparaiso, Chile; 3 School of Computer and Communication Sciences and Brain-Mind Institute, Ecole Polytechnique Fédérale de Lausanne, 1015 Lausanne EPFL, Switzerland; 4 INSERM U846, Stem Cell and Brain Research Institute, Bron, France; University of Lyon, University of Lyon1, 69500, France

## 

While monkeys perform a task alternating between behavioral adaptation --relying on feedback monitoring and memory of previous choices-- and repetition of previous actions, firing rates in dorsal Anterior Cingulate Cortex (dACC) modulate with cognitive control levels [1]. Further, it has been hypothesized that dorsolateral Prefrontal Cortex (dlPFC) could make use of dACC signals to take the adapted decision [2]. However, little is known about whether dACC spike timing may participate to behavioral adaptation signaling, and how multiple unit activities may be jointly decoded by dlPFC. We tested the hypothesis that temporal (or spatiotemporal) pattern matching mediate information transmission by dACC.

We used spike-train metrics [3] to decode dACC activity recorded in a problem solving task [1] (336 cells, 2 monkeys). We computed a similarity measure between every spike train and the activity patterns in different task epochs for classification. When computing the similarity, we explored different degrees of (i) timing sensitivity and (ii) distinction between spikes from different neurons, which led to different classification performances.

We found that timing sensitivity could improve behavioral adaptation vs. repetition classification of single unit spike trains. Optimal decoding occurred when accounting for spike times at a resolution <= 200 ms. Furthermore, spike-train metrics decoding of unitary discharges was related to the monkeys' response time. A downstream neural decoder could exploit this temporal information through coincidence detection determined by synaptic and membrane time constants. In addition, when decoding two units jointly, we found that each pair had a specific optimal distinction degree between spikes coming from the two different neurons. In a realistic neural decoder, the tuning of this distinction degree might occur through non-linear dendritic integration.

To further investigate the computational properties of temporal decoding in the context of decision-making, we are implementing a recurrent spiking neural network with connectivity leading to attractor dynamics. In this framework, each decision is mapped to a state in which a corresponding neural subpopulation shows elevated activity, as observed experimentally in dlPFC [4]. In addition, neurons will undergo membrane properties adaptation [5] and short-term plasticity, leading to history-of-choices dependent temporal spike patterns. We will investigate how this temporal-spike-patterns memory of past choices could interact with dACC feedback-specific temporal patterns to lead to adapted decision. The feasibility and putative advantages of spike-timing-dependent-plasticity-based learning of response selectivity to the appropriate dACC-dlPFC temporal correlations will be considered.

## Conclusions

The spatiotemporal structure of spike trains appears to be relevant in this cognitive task. This opens the possibility for pattern matching-based decoding of dACC activity, potentially leading to adapted behavioral response.
